# Intraocular pressure after myopic laser refractive surgery measured with a new Goldmann convex prism: correlations with GAT and ORA

**DOI:** 10.1186/s12886-022-02309-x

**Published:** 2022-02-16

**Authors:** María Iglesias, Bachar Kudsieh, Andrea Laiseca, Cristina Santos, Jeroni Nadal, Rafael Barraquer, Ricardo P. Casaroli-Marano

**Affiliations:** 1 Instituto Universitario Barraquer, Barraquer Ophthalmology Centre, Laforja 88, 08012 Barcelona, Spain; 2 Department of Ophthalmology, Hospital Universitario Puerta De Hierro, 28222 Madrid, Spain; 3 Unitat Antropologia Biològica, Department Biologia Animal, Biologia Vegetal i Ecologia, Universitat Autònoma de Barcelona (UAB), 08193 Barcelona, Spain; 4 International University of Catalunya (UIC), 08017 Barcelona, Spain; 5 Department of Surgery, School of Medicine and Hospital Clinic de Barcelona, University of Barcelona (UB), 08036 Barcelona, Spain

**Keywords:** Tonometry, Corneal biomechanics, Intraocular pressure, Glaucoma, Myopia, LASIK, PRK

## Abstract

**Background:**

The purpose of this study is to describe measurements using a newly developed modified Goldmann convex tonometer (CT) 1 year after myopic laser refractive surgery. Intraocular pressure (IOP) measurements were compared with IOP values obtained by Goldmann applanation tonometer (GAT), and Ocular Response Analyzer (ORA).

**Methods:**

Prospective double-masked study performed on thirty eyes of thirty patients that underwent laser in situ keratomileusis (LASIK; *n* = 19) or photorefractive keratectomy (PRK; *n* = 11). IOP was measured before and 3 and 12 months after surgery. Intraclass correlation coefficient (ICC) and Bland-Altman plot were calculated to assess the agreement between GAT, CT, IOPg (Goldmann-correlated IOP) and IOPcc (corneal-compensated IOP) from ORA.

**Results:**

Twelve months after LASIK, IOP measured with CT showed the best correlation with IOP measured with GAT before surgery (GATpre) (ICC = 0.886, 95% CI: 0.703–0.956) (15.60 ± 3.27 vs 15.80 ± 3.22; *p* < 0.000). However, a moderate correlation was found for IOP measured with IOPcc and CT 12 months after LASIK (ICC = 0.568, 95% CI: − 0.185 – 0.843) (15.80 ± 3.22 vs 12.87 ± 2.77; *p* < 0.004). Twelve months after PRK, CT showed a weak correlation (ICC = − 0.266, 95% CI: − 3.896 – 0.663), compared to GATpre (17.30 ± 3.47 vs 16.01 ± 1.45; *p* < 0.642), as well as poor correlation (ICC = 0.256, 95% CI: − 0.332 – 0.719) with IOPcc (17.30 ± 3.47 vs 13.38 ± 1.65; *p* < 0.182).

**Conclusions:**

Twelve months after LASIK, IOP measured with CT strongly correlated with GAT before surgery and could therefore provide an alternative method for measuring IOP after this surgery. More studies regarding this new convex prism are needed to assess its accuracy.

## Background

Laser in situ keratomileusis (LASIK) is the most popular corneal refractive surgery performed in the last decade [1], with an estimated one million myopic patients undergoing LASIK every year [2]. In view of this circumstance, it is expected that clinical practice will involve an increasing number of patients that have undergone laser refractive surgery (LRS) in the past.

It is known that corneal biomechanics (CB) are altered after LRS [3, 4]. The long-term results of postoperative visual acuity and the safety of this frequent procedure have been widely reported [5, 6]. Regardless of the surgery performed, CB can vary with age, which may affect corneal topography, visual outcomes and variations in tonometry measurements [7–10]. Corneal stiffness may increase with time due to a growth in glycogen-induced cross-links, and lead to different wound healing responses for PRK and LASIK [11, 12]. Moreover, biases in IOP measurements may lead to glaucoma misdiagnoses, especially in myopic eyes that are also known as a risk factor for developing open-angle glaucoma [13, 14].

**Fig. 1:**
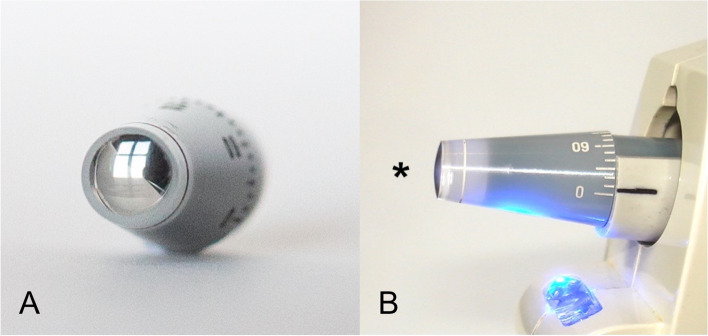
Modified CT applanation tonometer. Observe the convex reflection in the tip (**A**) and in the side image (B, *) CT tonometer inserted in a handling tonometer device (**B**)

The Ocular Response Analyzer (ORA) is a non-contact tonometer that is less influenced by CB modifications after LRS [3] than the Goldmann applanation tonometer (GAT), which is still the gold standard for measuring intraocular pressure (IOP) in normal corneas [15–17]. However, IOP underestimations in GAT measurements after LRS have been widely described [18, 19], due to changes in corneal central thickness (CCT) and CB [4, 20]. To overcome this underestimation of IOP in myopic eyes post LRS, we have developed a new method for measuring IOP in this subgroup of patients: the modified Goldmann convex tonometer (CT) (Fig. 1A) [4]. Using finite element analysis, we demonstrated that the applicability of the Imbert-Fick law is compromised after myopic LRS. When GAT reaches the 3.06 mm area of applanation, the flattened centre of an operated cornea (OC) is consistent with the idea of ​​Imbert-Fick behaviour, but not the corneal edges [4]. This indicates that there is lower resistance from the centre of an OC when we make physical contact with GAT, and as a result the IOP registered will be lower in these patients. Despite this, if a convex force is applied towards the centre of the ablated zone of an OC, a different phenomenon can be expected: initial contact pressure rises from the centre of the OC, resulting in a balance of forces comparable to that before surgery. By applying this force with the CT, we get to estimate the patient’s IOP from before their corneal procedure. In addition, this device functions exactly like GAT prisms and its measurements are reproducible by different observers [4], which implies that it is universal and simple to use, since it can be inserted in any slit-lamp or Perkins tonometer (Fig. 1B).

Recording pre-operative CCT and IOP measurements is very convenient for patient management after LRS [15] in addition to follow-up IOPm, taking into account that pre-operative measurement values do not remain stable throughout a patient’s life [4]. Given that IOP is the only risk factor in glaucoma progression that can be modified [21, 22], knowledge of the IOP baseline seems mandatory to create a prognosis profile [23, 24], even more so if LRS is not recent. Following our previous research in which we found a strong correlation between IOP using CT 3 months after myopic LASIK and GAT before surgery [4], we describe the clinical outcomes of this new Goldmann modified device in the long term, its correlations with GAT and with ORA by evaluating IOP measurements (IOPm) before and 3 and 12 months after myopic LRS.

## Methods

### Study design

A prospective, double-masked, single-centre, comparative study was carried out on a sample of thirty myopic subjects who were going in for femtosecond-assisted LASIK or photorefractive keratectomy (PRK) at the Barraquer Ophthalmology Centre in Barcelona. These subjects first underwent a general medical history review and a detailed eye examination. The study protocol adhered to the tenets of the Declaration of Helsinki and the centre’s Institutional Review Board approved this study. Written consent was obtained from all participants.

### Exploration protocol

All right eyes were randomly selected, adding up to a total of 30 eyes. The inclusion criteria for the study were: myopic Caucasian patients over 18 years of age with a stable myopic refractive error of less than − 9 spherical dioptres (dpts), and myopic astigmatism of less than − 4 dpts. Subjects with previously diagnosed ocular pathology, previous ocular surgery or receiving treatment with medications that may affect IOP levels were excluded. All patients underwent a standardised examination at baseline, 24 h, 1 week, 1, 3, and 12 months after surgery. To simplify comparisons in the long term, only 3 IOPm controls were considered for the study: at baseline and 3 and 12 months after surgery. These included measurement of visual acuity (spherical equivalent refraction, SE), slit-lamp anterior biomicroscopy, posterior segment ophthalmoscopy and IOPm. Age, gender, and refractive error were recorded. Before recording IOPm, corneal topography was carried out by Pentacam, (Oculus, Wetzlar, Germany), to obtain anterior simulated keratometry (simK) and CCT. Moreover, maximum ablation depth (Max.Abl) and percentage of ablated tissue (PTA) were analysed. Corneal characteristics including Corneal Hysteresis (CH), Corneal Resistance Factor (CRF), Goldmann-correlated IOP (IOPg) and corneal-compensated IOP (IOPcc) were also obtained by ORA (Reichert Ophthalmic Instruments, New York) after 3 different measurements with a waveform score (WS) higher than 3.5 as recommended [25], and the best score was selected.

In each patient, IOPm were taken between 10:00 and 13:00 h (10 am and 1 pm) [18] and ORA was performed at least 10 min before GAT [26] and CT measurements. Each applanation tonometer measurement was carried out with a double-mask protocol on the same slit lamp Goldmann tonometer, which is regularly calibrated by a certified assistant. The main observer (AL) measured IOP with different devices – GAT and CT – randomly given by a second observer (MI), and with 2-min rests between measurements to avoid tonographic effects [27, 28].

### Statistical analysis

Statistical analysis was performed using SPSS® (Statistical Package for the Social Sciences, version 22.0; SPSS Inc., Chicago, IL, USA), with the exception of the Bland-Altman analysis, for which *jamovi* 1.2 (jamovi project, 2020) was used. A nonparametric test was used due to the small sample size, and a significance level of 5% was considered in all analyses. We conducted descriptive analyses for all variables before surgery and 3 and 12 months after surgery. Descriptive values are presented as mean ± standard deviation (MD ± SD) unless stated otherwise. Comparisons between preoperative and postoperative values were performed using the Wilcoxon test, and comparisons between LASIK and PRK patients were performed using the Mann-Witney test.

GATpre was considered the current reference, and its correlation with IOPm using different tonometers was evaluated by: i) calculating the mean differences (md) between measurements with GATpre and all devices after surgery and testing for the absence of differences using the Wilcoxon test; ii) calculating the intraclass correlation coefficient (ICC) based on absolute agreement; values lower than 0.5, between 0.5 and 0.8, and greater than 0.8 were indicative of poor or weak, good, and excellent reliability, respectively [29]; and iii) constructing the Bland-Altman plot.

## Results

Thirty eyes of thirty patients were enrolled in the study and met the inclusion criteria in pre- and post-controls. Nineteen (63.3%) patients underwent LASIK and eleven (36.6%) PRK. The mean age was 30.7 ± 6.5 years. Table 1 shows descriptive statistics of the normally distributed variables in the pre- and post-surgical evaluations.

**Table 1:** Descriptive statistics of variables in the evaluations before and 3 and 12 months after surgery

	PRE-SURGERY MD ± SD	POST 3 M MD ± SD	POST 12 M MD ± SD
GAT (mmHg)^a^			
• ALL	15.80 ± 2.72	12.00 ± 2.32	13.04 ± 2.55
• LASIK	15.60 ± 3.27	**11.01 ± 2.31**	**12.90 ± 2.64**
• PRK	16.01 ± 1.45	**13.05 ± 1.13**	**14.20 ± 2.27**
CT (mmHg)^a^			
• ALL	21.07 ± 4.35	15.80 ± 3.07	16.30 ± 3.34
• LASIK	21.05 ± 4.59	**14.05 ± 2.67**	**15.80 ± 3.22**
• PRK	22.00 ± 4.01	**17.09 ± 2.55**	**17.30 ± 3.47**
IOPcc (mmHg)^a^			
• ALL	15.89 ± 3.07	13.07 ± 2.52	13.06 ± 2.40
• LASIK	16.40 ± 2.93	**12.48 ± 2.20**	**12.87 ± 2.77**
• PRK	15.02 ± 3.26	14.08 ± 2.83	13.38 ± 1.65
IOPg (mmHg)^a^			
• ALL	15.49 ± 2.98	8.95 ± 2.79	9.62 ± 2.73
• LASIK	15.81 ± 2.77	**8.24 ± 2.57**	**9.62 ± 3.00**
• PRK	14.93 ± 3.37	**10.17 ± 2.87**	**9.61 ± 2.32**
SER (dpts)			
• ALL	− 4.98 ± 2.28	0.00 ± 0.30	0.02 ± 0.22
• LASIK	−5.83 ± 2.21	0.05 ± 0.33	0.03 ± 0.25
• PRK	−3.52 ± 1.33	0.05 ± 0.23	0.03 ± 0.15
simK			
• ALL	43.70 ± 1.13	39.80 ± 1.96	39.90 ± 2.00
• LASIK	43.70 ± 1.16	39.30 ± 2.06	39.40 ± 2.04
• PRK	43.60 ± 1.11	40.60 ± 1.53	40.08 ± 1.62
CCT (μ)			
• ALL	543 ± 27.08	457 ± 35.90	469 ± 41.30
• LASIK	554 ± 22.30	460 ± 40.70	466 ± 42.40
• PRK	524 ± 26.40	452 ± 26.60	473 ± 41.10
CH			
• ALL	10.53 ± 1.28	7.88 ± 1.16	8.07 ± 1.03
• LASIK	10.44 ± 1.12	7.85 ± 1.10	8.04 ± 1.05
• PRK	10.67 ± 1.56	7.92 ± 1.32	8.11 ± 1.05
CRF			
• ALL	9.88 ± 1.12	6.29 ± 1.40	6.59 ± 1.25
• LASIK	9.97 ± 1.09	6.05 ± 1.37	6.54 ± 1.20
• PRK	9.72 ± 1.21	6.70 ± 1.43	6.68 ± 1.39
PTA			
• ALL		12.90 ± 4.73	
• LASIK		14.40 ± 4.94	
• PRK		10.30 ± 2.99	
Max.Abl			
• ALL		70.50 ± 26.80	
• LASIK		79.90 ± 27.50	
• PRK		54.10 ± 16.40	

Significant differences between LASIK and PRK IOPm are marked in bold

Comparisons between pre- and post-surgery: ^a^, Wilcoxon test, *p* < 0.001. All values are in mean ± standard deviation (MD ± SD). *GAT* Goldmann applanation tonometer, *CT* Convex tonometer, *IOPcc* Corneal-compensated IOP, *IOPg* Goldmann-correlated IOP, *SER* Spherical equivalent refraction, *simK* simulated keratometry, *CCT* Central corneal thickness, *PTA* Percent tissue altered, *Max.Abl* Maximum corneal ablation, *CH* Corneal hysteresis, *CRF* Corneal resistance factor, *M* Months

**Fig. 2:**
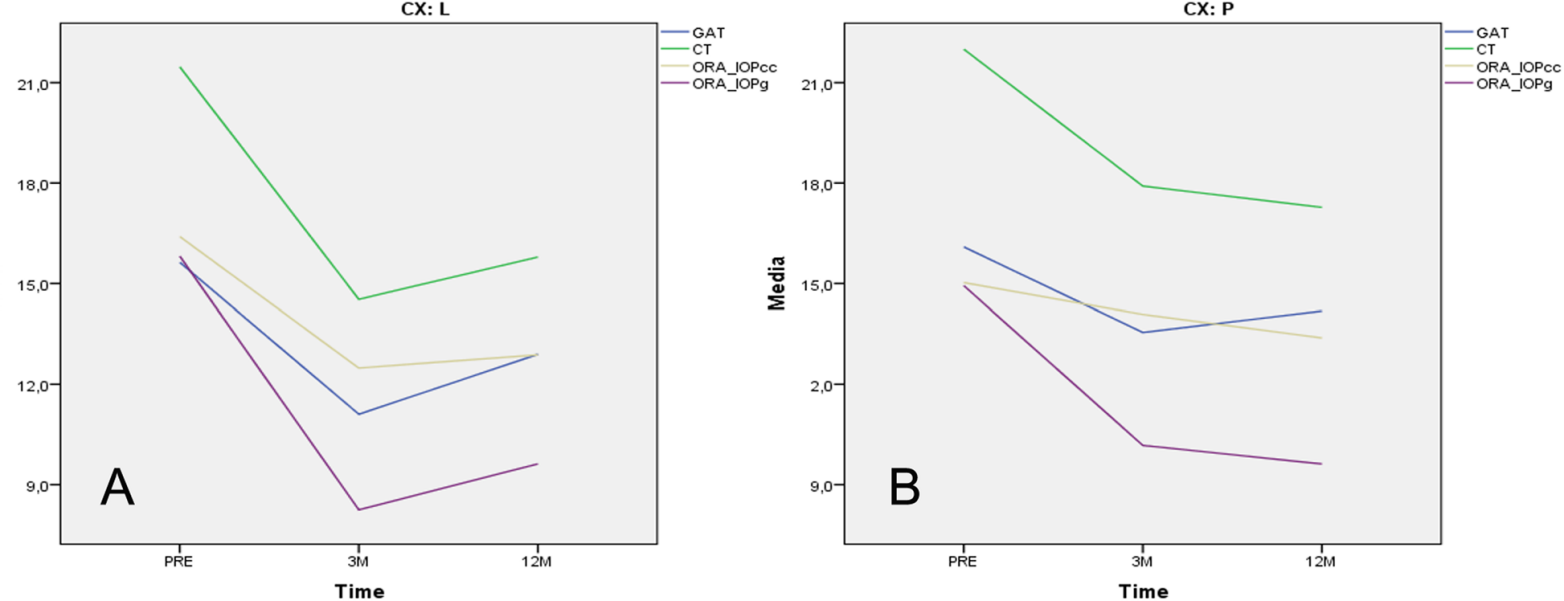
Mean IOP changes over time obtained using different tonometer devices in pre- and post-refractive surgery evaluations after 3 and 12 months. CX: L, LASIK (**A**); CX:P, PRK (**B**); GAT, Goldmann applanation tonometer; CT, convex tonometer; IOPg, Goldmann-correlated intraocular pressure and IOPcc, corneal-compensated IOP. M, months

Before surgery, ORA and GAT IOPm where similar between groups. In contrast, CT overestimated IOP in relation to the other tonometers. After surgery, all tonometers significantly registered lower IOP values, except for IOPcc in the PRK subgroup, with IOPg recording the lowest compared to GATpre (Table 1). In the LASIK subgroup after 3 months (Fig. 2A), GAT and IOPcc underestimated IOP, whereas CT at 3 months provided similar values to those obtained with GATpre and ORA. After 12 months, CT values were like GAT and ORA before surgery, whereas the rest of the tonometers kept IOPm below its pre-surgery estimations (Fig. 2A).

In the PRK subgroup (Fig. 2B), an IOP reduction was also found for all devices at 3 and 12 months after surgery. However, in this case, CT overestimated pre-surgery GAT, IOPg and IOPcc values; and IOPcc significantly registered the most stable IOPm compared to pre-surgery values.

The ICC, calculated for all patients and considering LASIK and PRK separately, presented poor or moderate correlation between GAT measurements before and after surgery (Table 2). The best correlations were observed in the LASIK subgroup between GATpre and CT at 3 months (15.60 ± 3.27 vs 14.05 ± 2.67; ICC = 0.808, 95% CI: 0.429–0.927; *p* < 0.000); and at 12 months (15.60 ± 3.27 vs 15.80 ± 3.22; ICC = 0.886, 95% CI: 0.703–0.956; *p* < 0.000). Moderate correlation was found for CT and IOPcc at 12 months (15.80 ± 3.22 vs 12.87 ± 2.77; ICC = 0.568, 95% CI: − 0.185 – 0.843; *p* < 0.004) (Table 2). The PRK subgroup – compared to GATpre – showed poor correlation for GAT and CT at 3 months (16.01 ± 1.45 vs 17.09 ± 2.55; ICC = − 0.196, 95% CI: − 1.817 – 0.619; *p* < 0.638) and also 12 months after surgery (16.01 ± 1.45 vs 17.30 ± 3.47; ICC = − 0.266, 95% CI: − 3.896 – 0.663; *p* < 0.642). A weak correlation was also found between CT and IOPcc at 3 and 12 months in the PRK subgroup (17.09 ± 2.55 vs 14.08 ± 2.83; ICC = 0.272, 95% CI: − 0.328 – 0.729; *p* < 0.116), (17.30 ± 3.47 vs 13.38 ± 1.65; ICC = 0.256, 95% CI: − 0.332 – 0.719; *p* < 0.182) respectively (Table 2).

**Table 2:** Intraclass correlation coefficient (ICC) results

	Tonometer pair	N	ICC (95% CI)	*P*
All patients	GAT3M - GATpre	30	**0.400** (− 0.227–0.741)	< 0.001
	GAT12M - GATpre	30	**0.478** (− 0.113–0.758)	< 0.006
	CT 3 M - GATpre	30	**0.669** (0.296–0.843)	< 0.002
	CT 12 M - GATpre	30	**0.704** (0.383–0.859)	< 0.001
	IOPcc3M- CT3M	30	**0.450** (− 0.136–0.740)	< 0.010
	IOPcc12M - CT12M	30	**0.465** (− 0.196–0.764)	< 0.003
LASIK	GAT3M - GATpre	19	**0.418** (−0.221–0.781)	< 0.003
	GAT12M - GATpre	19	**0.516** (−0.171–0.811)	< 0.015
	CT 3 M - GATpre	19	**0.808** (0.492–0.927)	< 0.000
	CT 12 M - GATpre	19	**0.886** (0.703–0.956)	< 0.000
	IOPcc3M- CT3M	19	0.405 (−0.252–0.748)	< 0.076
	IOPcc12M- CT12M	19	**0.568** (−0.185–0.843)	< 0.004
PRK	GAT3M - GATpre	11	0.134 (−0.234–0.590)	< 0.261
	GAT12M - GATpre	11	0.262 (0.581–0.752)	< 0.251
	CT 3 M - GATpre	11	−0.196 (−1.817–0.619)	< 0.638
	CT 12 M - GATpre	11	−0.266 (−3.896–0.663)	< 0.642
	IOPcc3M- CT3M	11	0.272 (− 0.328–0.729)	< 0.116
	IOPcc12M- CT12M	11	0.256 (− 0.332–0.719)	< 0.182

ICC is based on absolute agreement. Significant differences are marked in bold. CI, confidence Interval; *p,* value for correlation

*GAT* Goldmann applanation tonometer, *CT* Convex tonometer, *IOPg* Goldmann-correlated IOP, *IOPcc* Corneal-compensated IOP, *M* Months

Bland-Altman plots showed significant differences for GATpre and post measurements, and for CT and IOPcc after surgery. The mean differences for the entire cohort and between subgroups for IOPm before and after surgery can be found in Table 3. Considering LASIK separately, CT showed a small difference with GATpre at 3 months (md: − 1.11 mmHg, *p* = 0.440; LoA: − 3.32 – 5.53), the difference for CT at 12 months being the smallest (md: 0.158 mmHg, *p* = 0.438; LoA: − 5.58 – 5.26). On the other hand, CT showed small overestimations at 3 months in the PRK subgroup (md: 1.82 mmHg, *p* = 0.106; LoA: − 3.09 – 7.06) and at 12 months (md: 1.18 mmHg, *p* = 0.512; LoA: − 6.60 – 7.86). Additionally, CTpost significantly overestimated IOPccpost in LASIK and PRK respectively, at 3 months (md: 2.04 mmHg, *p* = 0.008; LoA: − 3.5 – 7.58) (md: 3.83 mmHg, *p* = 0.008; LoA: − 3.05 – 7.74), and 12 months (md: 2.91 mmHg, *p* < 0.000; LoA: − 2.53 – 8.35) (md: 3.89 mmHg, *p* = 0.03; LoA: − 2.95 – 8.04) respectively (graphics not shown).

**Table 3:** Mean differences for IOP measurements in pre-surgery with GAT and at 3 and 12 months after surgery with GAT, CT and IOPcc

	IOP difference	GAT3M - GATpre	GAT12M - GATpre	CT3M - GATpre	CT12M - GATpre	CT3M -IOPcc3M	CT12M -IOPcc12M
All	md (bias)	**−3.80**	**−2.43**	−0.0333	0.533	**2.70**	**3.27**
	Wilcoxon test	Z = -4.747;*p* < 0.000	Z = -3904;*p* < 0.000	Z = -0.361;*p* = 0.718	Z = -0.888;*p* = 0.375	Z = -3.754;*p* < 0.000	Z = -4.618;*p* < 0.000
LASIK GV	md (bias)	**−4.52**	**−2.74**	−1.11	0.158	**2.04**	**2.91**
	Wilcoxon test	Z = -3.836;*p* < 0.000	Z = -3.209;*p* < 0.001	Z = -2.017; *p* = 0.440	Z = -0.776;*p* = 0.438	Z = -2.636; *p* = 0.008	Z = -3.582;*p* < 0.000
PRK	md (bias)	**−2.54**	**−1.91**	1.82	1.18	**3.83**	**3.89**
	Wilcoxon test	Z = -2.185;*p* < 0.005	Z = -2.203;*p* = 0.028	Z = -1.616;*p* = 0.106	Z = -6.55;*p* = 0.512	Z = -2.667;*p* = 0.008	Z = -2.936;*p* = 0.03

Mean differences between GATpre, GAT, IOPcc and CT after surgery (at 3 and 12 months) were calculated and tested for the absence of differences using the Wilcoxon test. Significant differences are marked in bold. md, mean difference; Z, Wilcoxon test; *p,* probability value

*GAT* Goldmann applanation tonometer, *CT* Convex tonometer, *IOPg* Goldmann-correlated IOP, *IOPcc* Corneal-compensated IOP, *M* Months

## Discussion

Tonometry nowadays has become a complex concern since a single tonometer cannot be referred universally for every CCT, nor can CB and prior surgical procedures such as LRS or transplants be dismissed when measuring IOP [30, 31].

A reduced CCT is not only an independent factor for developing glaucoma in the future [32], but also challenge for estimating IOP if such reduction is due to a laser-assisted procedure, regardless of the device used to obtain IOPm. It is known that after laser ablation, thicker corneas preserve more biomechanical properties than thinner corneas [33, 34], and high myopia has a greater decrease in CH and CRF properties after LASIK due to greater anterior flap stroma lamellae reduction [35]. Touboul et al. described that the lower the CH, the lower the IOP underestimation with GAT, defining CH as a risk factor for underestimating IOP [33]. Taking into account that CH is lower in glaucoma [21, 36, 37] and myopic LRS patients [20, 38], GAT should be avoided in this subgroup of patients since they will have low CH values regardless of glaucoma. Therefore, seems crucial to focus on using only specific tonometers with these patients.

This convex prism has already been tested 3 months after LRS in comparison with GAT, registering higher IOPm in PRK than in LASIK subjects (1.62 ± 2.65 mmHg vs − 0.19 ± 2.60 mmHg, respectively) [4], concurring with the third month outcomes of the present study (1.82 ± 2.55 mmHg vs − 1.11 ± 2.67 mmHg). Still, this new modified prism has never before been compared in the long term. In a global overview of our measurements, IOPm at 12 months were slightly higher than IOPm at 3 months. This could be related to a partial recovery of CB after this period. Nonetheless, this assumption must be interpreted with caution, since the main limitation of the present study is that the number of patients undergoing measurements is not appropriate for estimating significant IOP correlations with CB or biological factors such as age or degree of myopia. Nevertheless, our CTpost LASIK results concur with the outcomes referred to above, where a significant correlation with CRF and CH reduction was found between CTpost and GATpre in 73 patients 3 months after LASIK (0.15 ± 3.22 vs − 0.19 ± 2.60 mmHg, respectively) [4]. Likewise, despite our small sample in the PRK subgroup, our outcomes in the current study are comparable with those of our previous publication: we measured 29 PRK patients after 3 months and the IOP deviation was higher than in the LASIK subgroup [4], confirming that we have enough evidence to assure that the modified Goldmann CT applicability in PRK patients and in standard corneas is not reliable due to preservation of CB. Further studies in larger cohorts will focus on determining if this convex prism performs accurately in LASIK patients.

Biases in estimating IOP may come from diverse sources, like introducing different devices in our daily clinical routine or determining inaccurate IOPm for a same patient [15, 39]. To minimize the biases of GAT post LRS, we can use non-contact tonometers such as the ORA, the non-contact tonometer (NCT), and the Corvis ST; or contact tonometers like the Pascal Dynamic Tonometer (PDCT) [40]. Among these, bIOP (biomechanical corrected IOP) from Corvis ST has shown to be the most stable and accurate parameter after surface ablation or lamellar procedure [39, 41]. Nonetheless, ophthalmologists do not always have access to devices that evaluate post-operative IOP accurately. In applanation tonometry after myopic LRS, IOP estimations must be considered carefully since they do not seem to follow CCT reduction linearly due to CB modifications. We believe it could be related in part to that contact pressure profiles between applanation tonometry and the anterior corneal surface are very different in PRK compared to LASIK patients [4]. Since less corneal tissue is removed from the anterior stroma in low myopia, CB are expected to be less altered in PRK, and therefore these corneas may behave like non-OC. In this study, we have compared a non-contact tonometer with two contact tonometers. Considering all of them together, we have found a significant IOP decrease in the LASIK subgroup compared to PRK. These findings coincide with recent results for other devices. Chen et al. measured IOP after 3 months with GAT, ORA, PDCT and Corvis ST [41], and also found a significant IOP reduction in the GAT and ORA subgroups after femtosecond laser-assisted LASIK compared to TransPRK. In addition, Schallhorn et al., in a larger cohort of LASIK and PRK patients measured with NCT [34], found a higher reduction of IOP after 3 months of LASIK (− 4.57 ± 2.42 mmHg), compared to PRK (− 3.16 ± 2.53 mmHg). In our previous research, despite a strong correlation found between CT and LASIK compared to PRK, GAT IOPm were significantly lower in the first group (− 3.94 ± 2.17 mmHg vs − 2.62 ± 2.16 mmHg) [4].

This is the first time that this new convex prism is being compared to ORA IOPm. ORA has been proven to improve GAT measurements by introducing new parameters that can evaluate CB modifications and IOP after LRS [3, 42]. IOPcc is known for being less influenced by CCT variations, compared to the IOP underestimation of IOPg and GAT post LRS [3, 43, 44]. Mean differences between GAT and IOPcc readings in standard corneas are ±1.5 mmHg [45]. However, despite GAT and IOPcc show higher correlations than IOPg [26, 46], they should not be used interchangeably because there can be differences of more than 2 mmHg between them which could have clinical implications [45]. In our sample, although a significantly good correlation was found between IOPcc and CT in the LASIK subgroup after 1 year – compared to a very poor correlation in the PRK subgroup –, mean differences were significantly higher than > 2 mmHg in both groups. Moreover, even though IOPcc has shown better agreement with GAT than with IOPg before LRS, and is more stable after surgery [26, 45, 46], we believe that an agreement between the convex prism and IOPcc cannot be expected after LRS. To begin with, CTpost has already demonstrated bad agreement with GATpost [4]; besides, in our study, CTpost overestimated IOPcc and IOPg values at 3 and 12 months, having similar correlations with GAT and IOPcc after 1 year, but those correlations were weak compared to correlations with GAT before surgery. This could be associated to the fact that matching static with dynamic tonometry seems to be incongruous since it has been widely proven that comparing these devices is not accurate [26, 46, 47]. An exact comparison must not be extrapolated considering that the central areas from which the IOP is measured are different between them: in applanation tonometry after LRS, both GAT and CT contact pressure profiles are not consistent with 3.06 mm and could encompass a larger amount of tissue especially in LASIK [4] compared to the exact central corneal 3.0 mm diameter from which ORA estimates IOP, regardless of the surgical procedure performed [48, 49]. These results suggest that the three tonometers should not be used interchangeably after LRS due to clinically significant IOP variations between them.

Another important limitation affecting our work, is that modified Goldmann CT measurements do not apply for every OC and may not represent real IOP, in the same way that GAT is not suitable for every non-OC, except for standard corneas with CCT around 520 μm (microns) and accurate measurement technique [50]. Since true IOP can only be obtained from invasive intracameral readings [43, 51], and IOPm may have not been taken at the same time of day after several months entailing IOP fluctuations between measurements [52], IOP stability after LRS was taken as a reference for the quality of tonometer readings [43]. In our study, IOPcc in the PRK subgroup was the most stable IOPm. It seems obvious to affirm that GAT and CT measurements before and after surgery are not reliable compared to themselves. However, as previously discussed, CT appears to be ineffective in non-OC, but its use is to register IOP after surgery in correlation to GAT before surgery. Nevertheless, CTpost mean differences between IOPm at 3 and 12 months were not significantly different, and they registered high correlations with GATpre values in the LASIK subgroup, concluding that convex prism IOPm in the sample presented may be considered acceptably stable after this procedure. A future study in larger LASIK populations will be carried out comparing the Goldmann CT and other tonometers to shed some light on its accuracy.

## Conclusions

In summary, though GAT is still the current reference technique in standard corneas, a certain IOPm variability is expected with applanation tonometry due to influencing corneal factors, especially after LRS [53, 54]. The new modified Goldmann CT is the first applanation tonometer to offer precise IOP estimations in LASIK patients after 3 [4] and 12 months in relation to GAT before surgery. It could be an affordable and effortless method for monitoring IOP by providing an additional reference to IOP assessment and therefore diminish the risk of glaucoma progression in this subtype of patients.

## Data Availability

The research data generated during the current study cannot be shared due to exclusive intellectual property rights of the new Goldmann “CT” convex tonometer secured by a Spanish patent filed with number P201631280 (granted patent), and a European patent filed with number 3520682 (granted patent). However, the data are available from the corresponding author upon reasonable request.

## Abbreviations

CB: Corneal biomechanics
CCT: Corneal central thickness
CH: Corneal hysteresis
CI: Confidence interval
CPP: Contact pressure profile
CRF: Corneal resistance factor
CT: Convexly-shaped tonometer
dpts: Dioptres
GAT: Goldmann applanation tonometer
ICC: Intra-class correlation coefficient
IOP: Intraocular pressure
IOPcc: Corneal-compensated IOP
IOPg: Goldmann-correlated IOP
IOPm: IOP measurements
LASIK: Laser-assisted in situ keratomileusis
LoA: Limits of agreement
LRS: Laser refractive surgery
M: Months
Max.Abl: Maximum ablation depth
md: Mean differences
MD ± SD: Mean ± standard deviation
NCT: Non-contact tonometer
OC: Operated cornea
ORA: Ocular Response Analyser
*p*: Probability value
PDCT: Pascal Dynamic Tonometer
PRK: Photorefractive keratectomy
PTA: Percentage of ablated tissue
SER: Spherical equivalent refraction
simK: Simulated keratometry
Z: Wilcoxon test
